# Towards reframing health service delivery in Uganda: the Uganda Initiative for Integrated Management of Non-Communicable Diseases

**DOI:** 10.3402/gha.v8.26537

**Published:** 2015-01-05

**Authors:** Jeremy I. Schwartz, Ashley Dunkle, Ann R. Akiteng, Doreen Birabwa-Male, Richard Kagimu, Charles K. Mondo, Gerald Mutungi, Tracy L. Rabin, Michael Skonieczny, Jamila Sykes, Harriet Mayanja-Kizza

**Affiliations:** 1 Uganda Initiative for Integrated Management of Non-Communicable Diseases, Kampala, Uganda; 2 Section of General Internal Medicine, Department of Internal Medicine, Yale University School of Medicine, New Haven, CT, USA; 3 Global Health Corps, New York, NY, USA; 4 Department of Community Health, Government of Uganda Ministry of Health, Kampala, Uganda; 5 Mulago National Referral Hospital, Kampala, Uganda; 6 Yale Global Health Leadership Institute, New Haven, CT, USA; 7 Department of Medicine, Makerere University College of Health Sciences, Kampala, Uganda

**Keywords:** Non-communicable diseases, Health system strengthening, Integration, Multi-sectoral collaboration

## Abstract

**Background:**

The burden of non-communicable diseases (NCDs) in low- and middle-income countries (LMICs) is accelerating. Given that the capacity of health systems in LMICs is already strained by the weight of communicable diseases, these countries find themselves facing a double burden of disease. NCDs contribute significantly to morbidity and mortality, thereby playing a major role in the cycle of poverty, and impeding development.

**Methods:**

Integrated approaches to health service delivery and healthcare worker (HCW) training will be necessary in order to successfully combat the great challenge posed by NCDs.

**Results:**

In 2013, we formed the Uganda Initiative for Integrated Management of NCDs (UINCD), a multidisciplinary research collaboration that aims to present a systems approach to integrated management of chronic disease prevention, care, and the training of HCWs.

**Discussion:**

Through broad-based stakeholder engagement, catalytic partnerships, and a collective vision, UINCD is working to reframe integrated health service delivery in Uganda.

Chronic non-communicable diseases (NCDs) are responsible for most of the world's morbidity and mortality (1). Over the next 15 years, the global financial losses from NCDs are expected to reach 7 trillion US dollars (2). This massive figure is driven by the fact that, in low- and middle-income countries (LMICs), NCDs disproportionately affect people in their peak wage-earning years. In 2012, all World Health Organization (WHO) member states committed themselves to achieving a 25% reduction in premature mortality from NCDs by 2025. Following this WHO resolution, coined ‘25×25’, a Global Action Plan arose in which countries proposed voluntary targets to help them reach that goal (2, 3).

In sub-Saharan Africa (SSA), the burden of NCDs in terms of disability-adjusted life-years increased by 45% between 1990 and 2010 (4). Ongoing epidemics of infectious diseases in SSA present the region's under-resourced health systems with a double burden of disease (5). Health system strengthening efforts throughout SSA, often guided by foreign aid prioritization, have traditionally been vertically oriented, or disease-specific. This leads to fragmented health service delivery. However, addressing NCDs, which share common risk factors (such as tobacco use, excessive alcohol consumption, unhealthy diet, and physical inactivity) and socioeconomic determinants of health (such as poverty, lack of education, and urbanization), will require a shift away from these siloed programs and towards diagonally oriented, integrated health service delivery platforms (6).

Integration of services within a health system is complex. No generally accepted definition of integrated care exists, though the universal aims are to improve health outcomes, patients’ experience, and efficiency in the system (7). Other values of integrated care include patient-centeredness, patient empowerment, and a reduction of barriers to healthy lifestyles. All of these are qualities of a functional primary care system (8). In 2009, Maher and colleagues laid out a framework to enhance the primary care response to NCDs in LMICs using a public health approach. They based this upon their experience of scaling up services for chronic infectious diseases such as HIV and tuberculosis. The goal of this proposed framework is to ‘reduce the burden of morbidity, disability, and premature mortality related to NCDs’. It contains a package of interventions for quality care that includes: political involvement and commitment; case-finding among persons attending primary care services; standardized diagnostic and treatment protocols; regular drug supply; and systematic monitoring and evaluation. Each element of this package, the authors suggest, need be coupled with key operations for national implementation of the interventions (9). Other authors have called for integration of NCD services into the existing HIV infrastructure (10–12). There are many examples of embedding NCD screening and detection into existing infectious disease-oriented programs (13–15). However, none of these options address the paradigm shift and redesign of the health system that will be needed to introduce successful primary care models in these countries.

In Uganda, NCDs have been among the Ministry of Health's (MoH) policy priorities since 2006 when the Programme for the Prevention and Control of NCDs was formed. Primary healthcare and integrated healthcare delivery are also MoH strategic priorities (16). Despite these policy priorities, implementation lags far behind due to inadequate local disease prevalence data, health services research, and financial resources to drive these policies. Following years of delay, the first nationally representative survey of NCD epidemiology— the WHO Stepwise approach to Surveillance (STEPS) survey— completed data collection in mid-2014. This survey will provide much needed epidemiologic information. A recent countrywide, NCD-focused needs assessment conducted by MoH sampled 54 health facilities. It revealed that the training and preparedness of Ugandan healthcare workers (HCWs) to diagnose and manage NCDs and the ability of the health system to cater to these patients is poor (17). Additionally, a recent analysis revealed that the national NCD Programme receives only 0.011% of the MoH budget and is funded largely from external grants (18).

In an effort to address the need for integrated approaches to health service delivery and HCW training in Uganda, we formed the Uganda Initiative for Integrated Management of Non-Communicable Diseases (UINCD). Herein we describe the process of UINCD's formation, the development of its mission and goals, challenges faced during the first year of operation, and current and proposed projects. Our hope is that a transparent portrayal in this manuscript of our formative processes will help guide the successful development of other similar multisector, international collaborations.

## Imagining a Center of Excellence for NCD integration

As part of an ongoing bidirectional medical education collaboration between Makerere University in Kampala, Uganda and Yale University in New Haven, CT (MUYU), colleagues from these institutions have worked together to provide clinical care and medical education at Mulago National Referral Hospital since 2006 (19). Together, they recognized the growing NCD burden among the population and the fragmentation of services for prevention and treatment. In 2011, these colleagues conceived the idea of a Center of Excellence (CoE) that would focus on NCD integration primarily through HCW training and health service delivery research. They proposed this idea to senior colleagues at both Makerere and Yale, and the concept was well received. The model for this concept was the Infectious Diseases Institute (IDI), a CoE also based at Mulago Hospital, that has been highly successful in addressing HIV and other common infectious diseases through patient care, HCW education, and research (20). There are many lessons from the HIV experience that are applicable to the NCD movement. They include, in part, the need to shift tasks between cadres of HCW, to develop robust clinical monitoring and evaluation programs, and to provide longitudinal, patient-centered care and medication adherence support (21). However, challenges unique to addressing NCDs include the diversity of complex disease processes despite common risk factors and the extended period of preclinical disease states requiring screening and early detection of modifiable risk factors. We recognized from the beginning of this process that addressing NCDs and their integration in the health services arena would require multisectoral collaboration and cooperation between diverse stakeholder groups.

## Broad-based stakeholder engagement

The next step in the process was the engagement of a broad spectrum of stakeholders. A stakeholder was defined as an individual or group with current or potential future involvement in NCDs in Uganda. Individual and organizational stakeholders were engaged via informal discussions. We began by speaking with colleagues in Makerere University College of Health Sciences. They recommended other individuals and organizations and the scope of engagement thereafter grew organically The stakeholders included individuals and organizations representing healthcare managers, academia, civil society organizations, non-governmental and governmental organizations, bilateral and multilateral institutions, and health informatics consultants (Table 1). Throughout this process, it was universally agreed upon that NCDs were in need of critical attention in Uganda, specifically focused on health services research, innovative HCW training models, and integration of services.

**Table 1:** Stakeholder groups engaged during formation and organization of UINCD

Initial round of engagement	Subsequent round of engagement
**Academia**	
Makerere University College of Health Sciences	
Department of Medicine, School of Medicine	Departments of Psychiatry and Surgery, School of Medicine
School of Public Health	
Medical Education Partnership Initiative-Cardiovascular Linked Award (MEPI–CVD)	
Infectious Diseases Institute	
**Government**	
Uganda Ministry of Health NCD Programme and Director General Clinical Services	Parliamentary Forum on NCDs
Mulago National Referral Hospital	
Uganda Cancer Institute	
Uganda Heart Institute	
Non-governmental organizations	
Uganda NCD Alliance	Uganda Muslim Medical Bureau
Uganda Heart Research Foundation	Uganda Catholic Medical Bureau
Uganda Diabetes Federation	Uganda Protestant Medical Bureau
Uganda Cancer Society	Management Sciences for Health
Danish Civil Society Fund	Center for Tobacco Control in Africa
	Alliance for Stroke Awareness and Prevention Project
**Bilateral/multilateral institutions**	
USAID	United Nations Development Program
WHO-Uganda NCD Country Advisor and Country Representative	
World Medical Association	
**Health informatics**	
OpenMRS software developer	

MEPI–CVD: Medical Education Partnership Initiative–Cardiovascular Disease Linked Award; NCD: non-communicable diseases; OpenMRS: Open Medical Record Software; USAID: United States Agency for International Development; WHO: World Health Organization.

## A catalytic partnership—the Yale Global Health Leadership Institute

The Yale-based partners in this effort approached the Yale Global Health Leadership Institute (GHLI). GHLI brings together policymakers, practitioners, and researchers who are collaborating with Yale faculty to foster evidence-based problem solving, inspire leadership, and debate critical issues in global health. The mission of GHLI is to develop leadership at Yale and around the world through education and research programs that strengthen health systems and promote health equity and quality of care. An annual conference held in New Haven, CT, convenes these multidisciplinary delegations to facilitate collaborative, locally driven solutions to improve health. GHLI found a number of features of this collaboration to be promising. The project was novel and would contribute new knowledge to the growing response to NCDs; it stood to have a substantial impact on health and be a model in other LMICs; and, given the established relationships and partnerships built upon years of collaboration in Uganda, there was a strong foundation for its success.

Aside from the annual conference, two additional elements promised to catalyze this project. For the first time in 2013, GHLI country delegations could compete for a $20,000 Innovation Award. Additionally, each country delegation would be paired with a GHLI student fellow. This fellow would work closely with the team during the conference and would return with the team members to their home country for 2 months to assist with initial implementation of the conference's output.

## Founding of UINCD

From the initial group of stakeholders, three specific criteria were used to select a core team of individuals to attend the 2013 GHLI Conference. First, the individuals collectively would represent diverse stakeholder groups. Second, they would be senior leaders from the highest levels of their representative stakeholder groups, thus being well-connected and well-positioned to effect change. Finally, the individuals would have similar levels of seniority within their respective institutions, thus enabling them to feel comfortable talking openly and debating with one another, and allowing for more progressive action.

The delegation from Uganda was comprised of leaders from the MoH NCD Programme, Mulago National Referral Hospital, Makerere University College of Health Sciences, and the Uganda NCD Alliance. The conference activities included faculty lectures on strategy development, stakeholder engagement, and the importance of leadership and culture; many hours of intensive, round-table work sessions during which GHLI consultants guided the Ugandan and Yale faculty partners through an eight-step strategic problem solving model; and a site-visit to the primary care CoE of the Veteran Affairs Medical Center in West Haven, CT (22).

The outcome of the Conference included the conceptualization and initial strategic plan for the UINCD. The delegation members (including the partners from Yale) formed the Steering Committee and agreed upon the following problem statement and mission as well as specific action items and key indicators for Year One (Table 2):

**Table 2:** Action items and key indicators for Year One of UINCD operations

Action items
Define UINCD and structure
Catalyze stakeholder buy-in
Establish project management structure
Launch UINCD with stakeholders
Develop and carry out HCW NCD needs assessment and evaluation plans
Cascade best practices to wider health system
Key indicators
UINCD structure in place
Needs assessments developed, tested, and completed
Quality improvement team established and active
Basic NCD curriculum for multidisciplinary health worker team training developed and rolled out

HCW: healthcare worker; NCD: non-communicable disease; UINCD: Uganda Initiative for Integrated Management of Non-communicable Diseases.

*Problem statement:* Ugandans do not receive integrated, evidence-based care along the entire NCD management spectrum.

*Mission:* To build capacity in the realms of prevention, clinical care, health worker training, and research to enable the provision of effective and integrated management of NCDs.

During and after the conference, a number of guiding principles emerged. First, UINCD should act as a nidus for the highest quality collaborative work focused on NCD integration and health system redesign. Second, UINCD should aim to carry out work that is cross-sectoral in nature, effective, locally relevant, implementable, and scalable.

Third, all of UINCD's activities should have a dissemination plan that includes local and regional media, stakeholders, and grey and/or academic literature. Finally, UINCD should, with regularity, reassess whether all of the right stakeholders are adequately engaged.

## Organization, partnerships, and initial projects

The newly formed UINCD Steering Committee decided to base its initial efforts at Mulago National Referral Hospital, with plans to eventually migrate towards research and implementation efforts in lower-level health centers and through community-based activities. We successfully competed for the GHLI Innovation Award and used these funds to support UINCD's initial research study and support a Program Manager. The organization was incorporated in Uganda as non-governmental organization.

The initial GHLI student fellow set the stage for close collaboration between UINCD and MoH. They designed and conducted the country's first needs assessment of public sector health facilities focused on NCD-related HCW knowledge and attitudes, clinical equipment, laboratory capabilities, and medication stocks (17). The findings of this yet-to-be-published study demonstrate that there is much need throughout the country and further strengthens the case for a critical reassessment and redirection of health service delivery in Uganda.

Two additional partnerships, with Global Health Corps (GHC) and TargetMobi, have proven to be important to UINCD's initial momentum. GHC brings together intelligent and passionate young professionals with organizations in LMICs and the United States in an effort to develop young leaders and to build a movement for health equity (23). UINCD was selected as a partner organization in 2014 and was paired with two fellows, one American and one Ugandan, who took on the respective roles of Curriculum Development Officer and Informatics Officer. Both fellows are charged with collaborating and engaging with UINCD stakeholder groups to advance cross-sector activities. For example, the Informatics Officer will work with an HIV partner organization and technology consultant to improve upon NCD-related data capture in the electronic medical record systems of HIV clinical environments. The Curriculum Development Officer will work with MoH and HIV partners to develop curricula that can be modified to the needs of various cadres of HCW. TargetMobi is a mobile research and communications technology company that enables researchers, healthcare providers, and businesses to incorporate mobile technology into their operations (24). In its partnership with UINCD, TargetMobi is programing UINCD's research study data collection instruments into tablet-based applications that sync with a cloud-based database. By removing manual data entry entirely, this will greatly simplify the data entry process.

UINCD's initial research study began at the end of the first year of operations. It is being conducted in the Assessment Center at Mulago Hospital, the main portal of entry for non-emergent patient care. This trial evaluates the effect of a series of simple interventions on providers’ management and triage of patients. The interventions aim to improve providers’ recognition of NCD risk factors such as tobacco, alcohol use, and obesity (by way of anthropometric measurements) and relevant clinical/diagnostic variables (blood pressure, blood glucose, and urine analysis) within the clinical encounter. The interventions include, among others, enhanced clinical record keeping, systematic collection of the above variables, and HCW training on an integrated approach to NCD management. The findings will inform UINCD's next steps as we work with our partners to study models of integrated healthcare delivery in various clinical and preclinical environments and develop targeted HCW training curricula that address NCDs alongside other important primary health issues.

The sustained commitment of UINCD's collaborators will set the project up for long-term success. The MoH probably represents the most critical collaborating organization in terms of providing prospects for implementation of UINCD's output. Support for UINCD was recently declared from the highest levels of MoH (25). Additionally, the commitment of human resources, financial, and in-kind support from our collaborators has been critical to our initial momentum and represents the dedication that will help make UINCD successful. Specifically, the UINCD Project Manager is also one of the Technical Officers in the MoH NCD Programme; clinical tools have been provided in-kind by MoH; MEPI-CVD has funded the initial UINCD training activities; and Mulago Hospital has provided office and clinical research space.

In October 2014, GHLI hosted its second conference for UINCD, this time in Kampala, Uganda. This ‘Forum for Change’ reconvened the Ugandan and U.S.-based members of the Steering Committee in order to take the next steps in goal-setting and developing the organizational structure. The Forum also brought together all of the initial stakeholder groups plus a large number of new potential collaborators to learn about UINCD's progress and discuss possible partnerships.

## Challenges and lessons learned during Year One

As multilateral research collaborations develop, the members need to pay attention to issues of equity between LMICs and high-income country (HIC) members (26). A number of important challenges have arisen over the first year that apply broadly to investigators engaging in academic global health collaborations. These challenges fall into five main categories: *Agenda-setting; Sharing academic credit; Funding; Stakeholder engagement; Balancing research and clinical care priorities*. Addressing these challenges has occurred through transparent, honest discussions at our group's meetings and in weekly phone calls (Table 3).

**Table 3:** Challenges faced in the first year of UINCD and the methods of addressing them

Challenge	Methods of addressing challenge
*Agenda-setting:* For a research collaboration such as UINCD, the issue of where the decision-makers are physically located and who sets the research agenda needs to be explicitly discussed.	• GHLI facilitated workshops on leadership and communication skills. • US- and Uganda-based collaborators have held weekly conference calls that help maintain open lines of communication and to bridge the physical distance. • The 2014 Forum focused on organizational structure, internal reporting, and a clearer description of individual roles and responsibilities.
*Sharing academic credit:* In a framework in which roles and responsibilities are shared, academic credit too must be shared. How do we explicitly negotiate these roles to maintain parity?	• Explicit discussions about authorship are had between collaborators at the outset of projects. • All protocols and research documents undergo joint review by US-based and Uganda-based co-investigators. • The research group strives for balance in authorship of documents, protocols, and grant applications.
*Funding:* In an equitable collaboration, through what channels does the funding stream? As granting agencies increasingly desire LMIC-based principal investigators (PIs), how do we maintain financial support for US-based collaborators?	• Though it remains unclear where UINCD will be most successful at finding funding, non-traditional sources of funding, such as in the Ugandan private sector, will need to be sought. • A diversification of funding streams must be pursued because funding options that only allow LMIC-based PIs pose a challenge to HIC-based investigators whose institutions place high value on the acquisition of grant dollars.
*Stakeholder engagement:* Work related to NCDs involves more stakeholders than disease-specific trials or epidemiologic studies. How do we balance the interests of multiple stakeholder groups and work together to translate scientific results into policy and systemic change?	• UINCD must think broadly about what defines a stakeholder group and specifically aim to include communities often left out of the NCD arena, such as those addressing injury/disability and mental health. • UINCD must engage with stakeholders early and re-engage with them often.
*Balancing research and clinical care priorities:* Is UINCD primarily focused on research or patient care?	• Recalling that the original concept of UINCD formed from observing patient care experiences and a desire to improve patient care. • Agreement that UINCD does not intend to set up a separate healthcare structure, but rather to test models of care that could be rolled out by partner organizations.

GHLI: Yale Global Health Leadership Institute; HIC: high-income country; LMIC: low- and middle-income country; NCD: non-communicable disease; PI: principal investigator; UINCD: Uganda Initiative for Integrated Management of Non-communicable Diseases.

## Conclusions

This is a case study of a fledgling research collaboration that brings together diverse stakeholder groups in a partnership focused on integrating NCDs throughout the Ugandan health sector. Though the goals of this collaboration are lofty and numerous challenges remain, we believe that UINCD will succeed because of a multiyear process of concept design and refinement built upon long-lasting relationships; broad stakeholder engagement; partnership development; and the incremental and strategic roll-out of projects. We hope this model of collaboration formation can be used to guide similarly minded endeavors in other countries.
